# Effectiveness and Safety of Oral* Cordyceps sinensis* on Stable COPD of GOLD Stages 2–3: Systematic Review and Meta-Analysis

**DOI:** 10.1155/2019/4903671

**Published:** 2019-04-03

**Authors:** Xuhua Yu, Yuquan Mao, Johannah Linda Shergis, Meaghan E. Coyle, Lei Wu, Yuanbin Chen, Anthony Lin Zhang, Lin Lin, Charlie Changli Xue, Yinji Xu

**Affiliations:** ^1^The Second Affiliated Hospital of Guangzhou University of Chinese Medicine, Guangzhou 510120, China; ^2^School of Health and Biomedical Sciences, RMIT University, Bundoora, VIC 3083, Australia

## Abstract

*Cordyceps sinensis* (CS) is a complementary medicine used for Chronic Obstructive Pulmonary Disease (COPD) of Global Initiative for Chronic Obstructive Lung Disease (GOLD) stages 2-3. Many randomized controlled trials have been conducted to evaluate the effect of CS alone or in combination with other herbs on stable COPD. To provide a synthesis of the evidence, we searched nine major electronic databases for randomized controlled trials on CS published before 21st December 2016. Fifteen interventional studies, including 1,238 participants, met the inclusion criteria. Meta-analysis showed that both CS preparations and CS formulae showed the potential benefits in lung function, exercise endurance, life quality, and improvement of symptoms. No serious adverse events were reported. So CS may be a promising treatment for patients with stable COPD of GOLD stages 2-3. No studies were placebo-controlled or of high methodological quality, which limits the conclusions.

## 1. Introduction

Chronic Obstructive Pulmonary Disease (COPD) is a common disease of the respiratory system, characterized by persistent airflow limitation. COPD is caused by the continued chronic inflammation when airways are exposed to cigarette smoke, noxious particles, or smoke from biomass fuels [1–3]. COPD has been a major cause of chronic morbidity and mortality throughout the world [4, 5], especially in developing countries.

Bronchodilators and inhaled corticosteroids are used to control disease progression and improve quality of life and are the most common pharmacotherapies for stable COPD. Both treatments have reported side effects, including increased heart rate with bronchodilators and increased risk of pneumonia with inhaled corticosteroid (ICS) [6] and ICS may increase risk of diabetes in COPD patients [7]. Unwanted side effects may lead some people with COPD to explore other treatment options.

*Cordyceps sinensis *(CS), Chinese name* Dong chong xia cao*, is a fungus parasitic on certain caterpillars. It has been in use in China for treatment of lung conditions since at least the 17th century, as described in the classical medical text Ben Cao Cong Xin [8]. Few side effects have been reported with CS [9], and no obvious toxic effects on hematology, liver, and kidney function and other organs have been found in rats and rabbits [10]. The instructions of some Chinese patent medicines with CS as the main ingredient only mentioned that a few patients had pharyngeal discomfort. Many clinical trials have been conducted on CS to evaluate its effect in people with GOLD stage 2-3 COPD, including studies of CS alone and in combination with other herbs as a formula. There are two systematic analyses reported on CS alone on stable COPD [11, 12] and they showed CS could improve lung function, exercise ability, life quality, and arterial partial pressure of oxygen.

In this study we searched electronic biomedical literature for studies of both CS alone and CS formulae on stable COPD of GOLD stages 2-3 and systematically reviewed its effectiveness. We conducted comparisons of CS preparations on lung function, exercise capacity, acute exacerbations, health-related quality of life, and effective rate. Further, we reviewed adverse events in included studies.

## 2. Method

### 2.1. Search Strategy

Following the methodology of the Cochrane Airways Group, we searched the following electronic databases without language restriction: PubMed, Cochrane Central Register of Controlled Trials (CENTRAL), Embase, Allied and Complementary Medicine Database (AMED), the Cumulative Index to Nursing and Allied Health Literature (CINAHL), China National Knowledge Infrastructure (CNKI), Chinese Scientific Journals Full text Database (CQVIP), China Biology Medicine Database (CBM), and Wanfang Database. Databases were searched to 21st December 2016.

Search terms were as follows: COPD, chronic obstructive pulmonary disease, COAD, chronic obstructive airway disease, chronic obstructive lung disease, chronic obstructive airway disease, airflow obstruction, chronic airflow obstruction, Jin Shui Bao, Bai Ling,* Cordyceps*,* Cordyceps sinensis*,* Cordyceps military,* and their MeSH synonyms. The names for two CS preparations were included in the search terms (Jin Shui Bao and Bai Ling). Synonyms of COPD or* Cordyceps* were combined with “OR” Boolean logic operator, and “AND” was used between COPD and* Cordyceps*.

### 2.2. Study Selection

*Inclusion Criteria*. (1) Randomized controlled trials (RCTs) in patients with GOLD stage 2-3 COPD: the criteria for GOLD stage 2-3 were postbronchodilator FEV1>30% and <80% predicted, according to Global Initiative for Chronic Obstructive Lung Disease in 2010 [1] or Guideline in China [13–15].

(2) Intervention: oral CS preparations and its formulae were categorized as follows: (a) preparations of CS, such as Bai Ling Capsule and Jin Shui Bao Capsule, in which fermented CS is the main component in the preparations besides other additional agents such as vitamin B1 and microelements and (b) CS formulae which combined CS with other herbs. Studies which used CS alone or in combination with routine care/pharmacotherapy were included.

(3) Controls: routine care or western medicine (WM) based on the guideline, nonpharmacological respiratory therapies, such as smoking cessation, pulmonary rehabilitation, oxygen therapy, and pulmonary exercise, was included and used in both the treatment and control groups. Studies which used a wait list control or no treatment in the control arm were also included.

(4) Outcome measures: the primary outcome measures were spirometric parameters (FEV1% predicted or FEV1/FVC ratio), six-minute walk distance (6MWD), St. George's Respiratory Questionnaire (SGRQ) total score, and frequency of exacerbation. Secondary outcome measures were effective rate, which is a global assessment of improvement in symptoms including dyspnea, mental status, appetite, and sweat according to chronic bronchitis section on the Guidance for Clinical Research on New Drugs of Traditional Chinese Medicine [16] and adverse events.

*Exclusion Criteria*. Studies which did not meet the above criteria were excluded. Additionally, the following studies were excluded: (1) CS alone or in a formula used in combination with other nonoral herbal interventions, such as Danshen injections; (2) CS alone or in a formula used in combination with other Chinese medicine therapies, such as Qi Gong or acupuncture; (3) studies that included participants with respiratory failure, pulmonary hypertension, or corpulmonale; (4) studies in which the treatments were not recommended for stable COPD, for example, the studies using Bacille Calmette-Guerin or antibiotics as routine care.

### 2.3. Data Extraction and Quality Assessment

Two authors (Yu Xuhua and Mao Yuquan) assessed studies independently based on the inclusion and exclusion criteria. Both authors extracted data for included studies and verified the accuracy of data. Where differences were identified, two reviewers (Charlie Xue and Xu Yinji) were consulted if necessary. Characteristics and outcome measures of the studies were tabulated and compared.

Risk of bias was assessed for all the included studies, and this work was done by two authors (Yu Xuhua and Mao Yuquan) independently according to the Cochrane risk of bias tool [17]. Disagreement on assessments was resolved through a third researcher (Wu Lei and Chen Yuanbin). To clarify unclear information in the included studies, study team members contacted the authors for additional details through telephone, email, or postal mail. While contact was made, additional information was not always available.

### 2.4. Evidence Synthesis

Data were analyzed by Review Manager version 5.3 [18]. Mean difference (MD) was used to evaluate continuous variables and odds ratio (OR) was calculated to evaluate dichotomous variables with 95% confidence intervals (CI). The results of the combined trials were calculated with random or fixed-effect model according to I^2^. Sensitivity analysis was used to explore heterogeneity. Subgroup analysis was also conducted for studies which used CS preparations and those which used CS as part of a formula. Publication bias was checked with funnel plots when more than five studies in each subgroup reported the same outcome measure [19].

## 3. Result

### 3.1. Description of Studies

**Figure 1:**
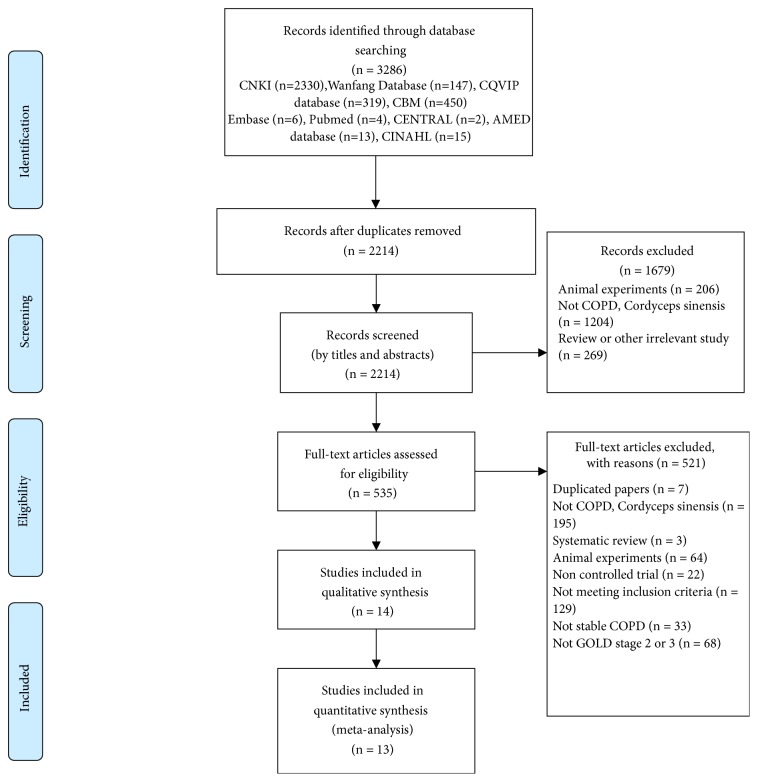
Flow chart of study selection.

Of 3,286 potentially relevant studies, 1,072 were excluded as duplications, and 1,679 were neither clinical studies, nor studies on CS, nor related to COPD based on scanning the titles and abstracts. The remaining 535 citations were selected for full article review. Seven were duplicated papers, 195 were unrelated papers (not CS or not including patients with COPD), 67 were not clinical research, and 252 did not meet with the inclusion criteria. Finally, 14 studies [20–33], including 1,192 participants, were included, and 13 studies including 1,092 participants [20, 22–33] were accessed by meta-analysis. The inclusion and exclusion process were summarized in Figure 1.

All of 14 studies were conducted in China, 12 studies [20–26, 28–31, 33] are journal articles and the other two are theses [27, 32]. Duration of COPD in all subjects ranged from 4.7 to 15.2 years, and treatment durations varied from one to 12 months. Of these trials, two reported the follow-up assessment at six months after end of treatment [31, 33]. Three studies reported loss to follow-up [20, 27, 32], and 1,169 participants were included in outcome assessment. The characteristics of the included studies are summarized in Table 1.

Six studies [21, 22, 24, 30, 31, 33] used oral capsules containing CS as the main Chinese herbal medicine intervention. The other eight studies [20, 23, 25–29, 32] used CS combined with other herbs. Thirteen studies [20–24, 26–33] compared the combination of CS and WM versus the same WM. The remaining citation [25] compared CS formula to nonpharmacological care control. Details of the interventions are reported in Table 2.

### 3.2. Methodological Quality

**Figure 2:**
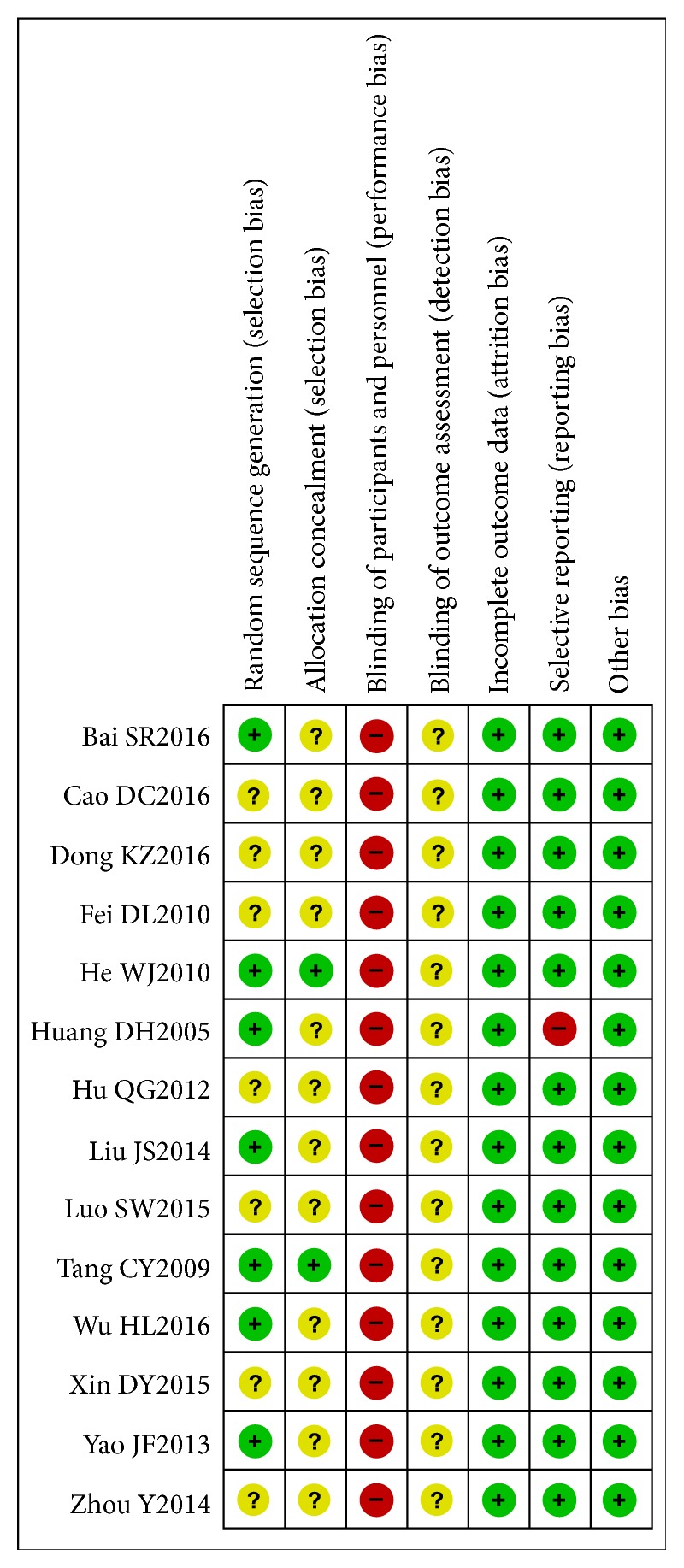
Risk of bias summary.

Risk of bias assessment is shown in Figure 2. All studies were described as “randomized”. Seven studies [20, 24, 26–28, 31, 32] described the method of sequence generation (by software or random number table) and were judged to be low risk of bias. Two studies [31, 32] concealed group allocation in an envelope, so they were judged as “low risk” for allocation concealment. All studies were at high risk of bias for blinding of participants and personnel because they were all open label studies. None of the studies described whether the outcome assessor was blind to group allocation, so all were judged as “unclear” in blinding of outcome assessment. One study by Huang DH [26] was judged as “high risk” for selective outcome because results for one outcome (acute exacerbations) were presented in a different way to that specified in the methods. All studies were at low risk for incomplete outcome data. Three studies [20, 27, 32] reported dropouts, and all provided adequate explanations. The rate of dropouts was lower than 15% (see Table 1), and based on the reasons provided, dropouts were not related to treatment received. All studies were judged as “low risk” for other sources of bias which included funding, conflicts of interest, and baseline balance.

### 3.3. Publication Bias

It was not possible to conduct funnel plot evaluation because the number of studies in each subgroup of outcome measures was less than five.

### 3.4. Outcome Measures

#### 3.4.1. Meta-Analysis of FEV1% Predicted

**Figure 3:**
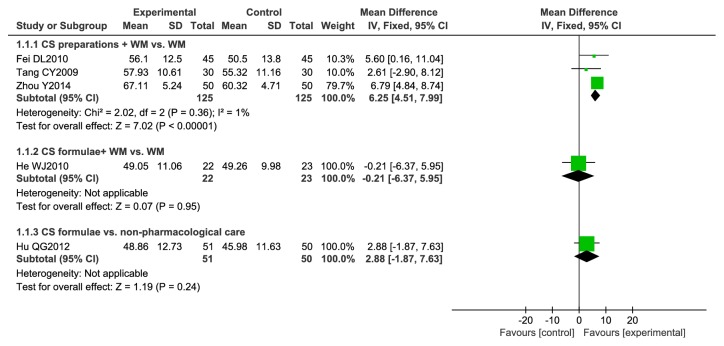
Meta-analysis of FEV1% predicted.

Analyses are showed in Figure 3. Five studies [22, 25, 31–33] reported FEV1% predicted (Figure 3), which were divided to three subgroups: (1) CS preparations plus WM vs. WM, (2) CS formulae plus WM vs. WM, and (3) CS formulae vs. nonpharmacological care. Subgroup 1 included three studies. The analysis result showed that the combination of CS preparations and western medicine increased FEV1% predicted by 6.25% (95% CI 4.51 to 7.99, I^2^ = 1%). Only one study was in subgroup 2 and subgroup 3, respectively. CS formulae combined with WM increased FEV1% predicted by -0.21% (95% CI -6.37 to 5.95). CS formulae combined with oxygen therapy increased FEV1% predicted by 2.88% (95% CI -1.87 to 7.63). Both CS formulae plus WM and CS formulae plus oxygen therapy did not show any effect on improving FEV1% predicted.

#### 3.4.2. Meta-Analysis of FEV1/FVC Ratio

**Figure 4:**
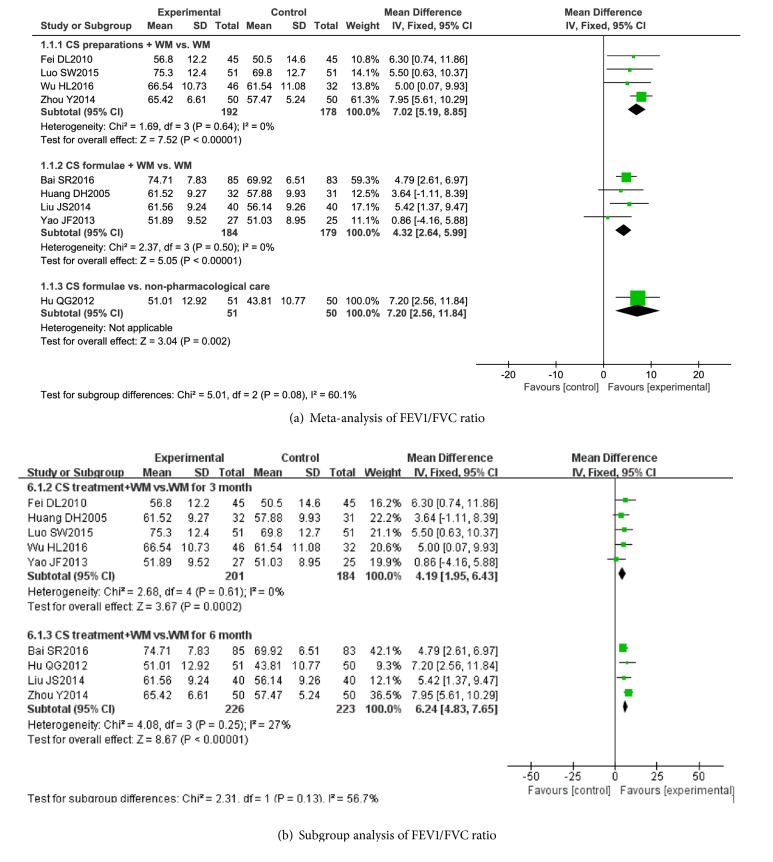


Analyses are shown in Figure 4. Nine studies [20, 22, 24–28, 30, 33] were included in analyses of FEV1/FVC ratio. The results showed that CS preparations combined with WM improved the ratio of FEV1/FVC by 7.02% (95% CI 5.19 to 8.85, I^2^ = 0%). Additionally, the combination of CS formulae and WM also showed improving of the ratio (MD 4.32%, 95% CI 2.64 to 5.99, I^2^ = 0%) when compared to the same WM. In a single study, CS formulae improved FEV1/FVC ratio by 7.2% compared with nonpharmacological care (95% CI 2.56 to 11.84).

Subgroup analysis of duration showed that CS treatment combined with WM for 3 months improved the ratio of FEV1/FVC by 4.19% (95% CI 1.95 to 6.43, I^2^ = 0%). When duration was longer than 6 months, CS treatment combined with WM improved the ratio of FEV1/FVC by 6.24% (95% CI 4.83 to 7.65, I^2^ = 27%).

#### 3.4.3. Meta-Analysis of 6MWD

**Figure 5:**
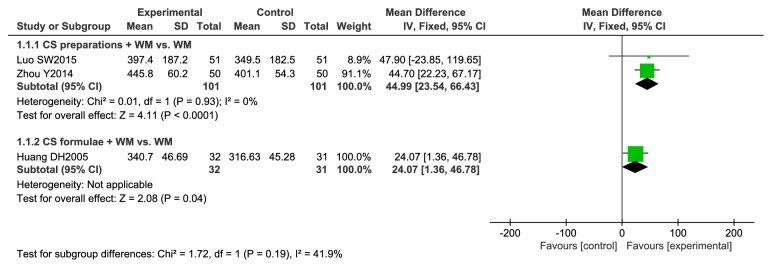
Meta-analysis of 6 MWD.

Analyses are showed in Figure 5. Three studies [26, 30, 33] were included. The subtotal effect indicated that the treatment improved mean distance walked in 6 minutes by 44.99 m (95% CI 23.54 to 66.43) more than controls when the CS preparations were combined with WM. Not only the result is statistically meaningful, but also the improvement is more than the minimum clinically important difference (MCID) (37-71 m) [34]. In the single studies, the effect of CS formulae combined with WM on 6MWD was better than WM alone (MD 24.07 m, 95% CI 1.36 to 46.78).

#### 3.4.4. Meta-Analysis of Outcome Measures for SGRQ Scores

**Figure 6:**
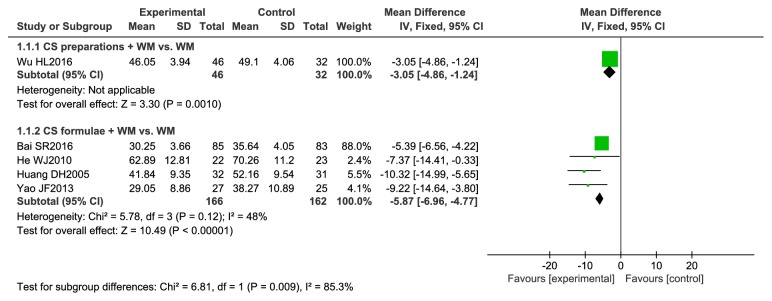
Meta-analysis of outcome measures for SGRQ scores.

Analyses are shown in Figure 6. Five studies [20, 24, 26, 27, 32] were included. A single study showed that CS preparations plus WM reduced SGRQ score by 3.05 points (95% CI -4.86 to -1.24) compared to WM. Four studies compared CS formulae plus WM with WM. The result showed that the SGRQ score was -5.87 points lower in people who received CS formulae combined with WM versus WM alone (95% CI -6.96 to -4.77, I^2^ = 48%). Lower scores indicate better quality of life. The reduction is clinically meaningful (MCID = 4 points).

#### 3.4.5. Meta-Analysis of Acute Exacerbation

**Figure 7:**
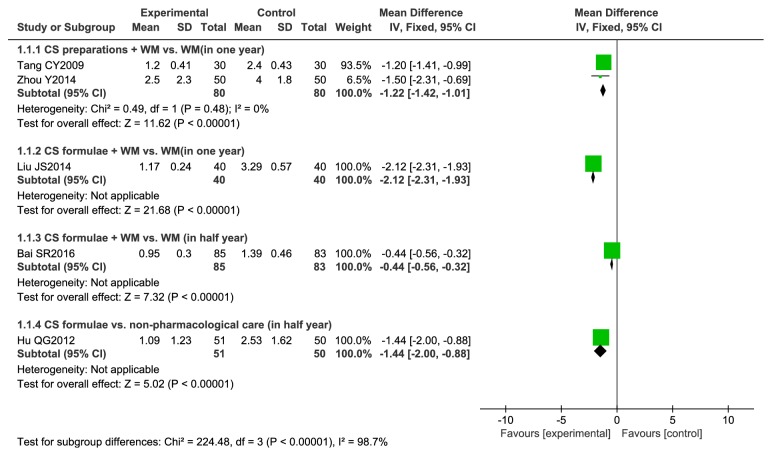
Meta-analysis of acute exacerbation.

Analyses are shown in Figure 7. Six studies [20, 25, 26, 28, 31, 33] reported on acute exacerbations. One study [26] reported data in a way that did not permit reanalysis following standard methods, and data were excluded from analysis. Five studies [20, 25, 28, 31, 33] were included for analysis. Three studies [28, 31, 33] reported frequency of exacerbations in one year, and two studies [20, 25] reported frequency of exacerbation in a half year. CS preparations combined with WM decreased the number of acute exacerbations by 1.22 events (95% CI -1.42 to -1.01, I^2^ = 0) per year. CS formulae plus WM resulted in 2.12 fewer exacerbations per year (95% CI -2.31 to -1.93) and 0.44 fewer exacerbations in a half year (95% CI -0.56 to -0.32) compared with WM alone. Compared with nonpharmacological care, CS showed a reduction of exacerbations by 1.44 events (95% CI -2.00 to -0.88) in a half year.

#### 3.4.6. Meta-Analysis of Outcome Measures for Effective Rate

**Figure 8:**
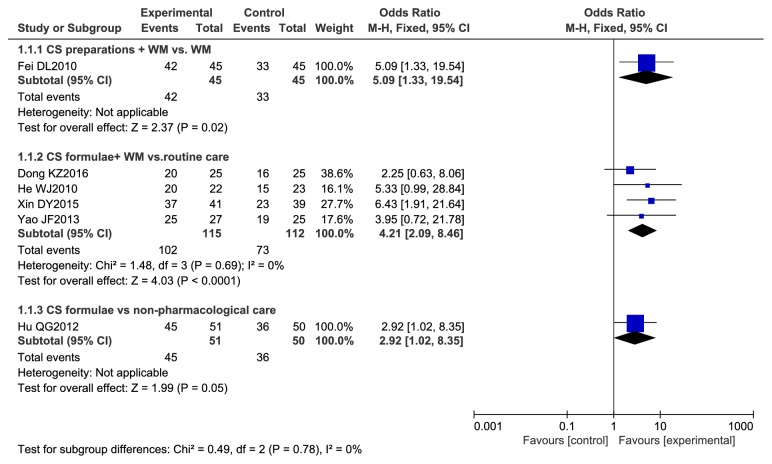
Meta-analysis of outcome measures for effective rate.

Analyses are shown in Figure 8. One single study [22] showed the combination of CS preparations and WM increased the odds ratio (OR) of effect rate by 5.09 times (95% CI 1.33 to 19.54) compared with WM alone. Four studies [23, 27, 29, 32] reported numbers of patients with symptom relieving when receiving CS formulae plus WM. Compared with patients not receiving CS, OR of effective rate for the patient receiving CS was higher (OR 4.21, 95% CI 2.09 to 8.46 I^2^ = 0%). CS formulae [25] increased OR of effective rate by 2.92 times (95% CI 1.02 to 8.35) compared with nonpharmacological care control.

#### 3.4.7. Adverse Events

Eight studies [21, 23–28, 35] reported on adverse events. One study [26] reported that two patients who received CS treatment had dry mouth and slightly abdominal distension, and one patient in the control group had loss of appetite. Another study [24] reported that two patients who received CS treatment had headache, abdominal distension, and felt uncomfortable in throat, and two patients in control group had abdominal distension and rash. No causality assessment was made for the adverse events. In the other studies, no adverse events were found.

## 4. Discussion

Systematic reviews and meta-analyses are at the top in the hierarchy of clinical evidence [36]. Many clinical trials have evaluated CS. This review synthesized the result from clinical trials to provide the best available evidence for CS for stage 2 to 3 COPD.

### 4.1. Main Findings

FEV1% predicted and FEV1/FVC ratio are not only the most important diagnostic criteria and classification criteria for COPD but also the important markers for disease progression. In this review, we found that CS alone combined with WM increased both FEV1% predicted and FEV1/FVC ratio. But the combination of CS formulae and WM did not improve FEV1/FVC ratio as much as CS alone. CS formulae showed a better effect on FEV1/FVC ratio compared to nonpharmacological care. Besides, subgroup analysis showed that treatment duration of CS formulae for more than 6 months improved FEV1/FVC ratio better than 3 months. It demonstrates that the effect of CS on FEV1/FVC ratio may be based on treatment duration and affected by the combined treatments (both herbs and WM).

Exercise tolerance reduction is another important characteristic of COPD. The reduced endurance is a result of cardiopulmonary function impairment and muscle wasting. Both CS alone and CS formulae showed the increased distance in six-minute walking. And the improvement was more than the MICD (37-71 m) [37] when the patients were treated by the combination of CS preparation with WM. This effect may be due to its antioxidant effect and antifatigue effect reported in the previous study [38].

Exacerbation is another concern in COPD treatment, as frequent exacerbation recurrence can worsen lung function. As such, the frequency of exacerbations is an important outcome measure. In this review, the CS alone and CS formulae showed higher odds ratios than the controls. This suggests that CS might improve the symptoms of stable COPD. However, the conclusions from studies which reported exacerbations in a half year are less reliable because of the impact of seasons [39].

SGRQ is a subjective criterion. It reflects the impact of the disease on a patients' health-related quality of life. CS preparations alone and CS formulae can reduce the SGRQ score and the changes in the SGRQ score exceeded the MCID of 4 points reported by Pellegrino [37].

Effective rate reflects changes in symptoms. The criterion is broadly used as a subjective outcome measure in Chinese traditional treatment. In this review, both CS alone and CS formulae could have a higher odds ratio than the controls. It shows that CS can improve the symptoms of stable COPD. This result is as a complementary outcome because it does not appear to have been validated.

Diversity was seen in formulation types, with most studies using CS capsules. Two CS manufactured products were used in multiple studies: Bai Ling capsule (8 studies) and Jin Shui Bao capsule (3 studies). Dosing was reasonably consistent, with the majority of studies reporting use three times daily. This is likely to be influenced by the large number of studies that used Bai Ling capsules. This meta-analysis found that CS preparations were more effective than CS formulae in improving FEV1% predicted. There was no clear evidence that one preparation type produced greater benefit than the other for other outcomes. Chinese medicine doctors may wish to consider patient preference for formulation type (capsules, pills or formulae) when prescribing treatment with CS.

Adverse-event reporting was incomplete and inconsistent among studies so we could not draw strong conclusions about adverse effects of CS.

### 4.2. Comparisons with Other Systematic Reviews Related to* Cordyceps sinensis*

Two systematic reviews [11, 12] have evaluated CS for COPD previously. One study was done by Mu Wei [12], which included 14 quasirandomized controlled trials published before 2011. The other systematic review [11] assessed the effect of Bai Ling capsule on FEV1, blood O_2_ pressure, and blood CO_2_ pressure of stable COPD patients. Different from the first review, trials in this review are all randomized controlled trials. In this review, the outcome measures include FEV1% predicted, FEV1/FVC, 6MWD, SGRQ score, exacerbations and effective rate, which are main outcome measurements to assess severity of stable COPD. The results for FEV1%, FEV1/FVC, 6MWD, and SGRQ are similar to the previous two reviews. This review included new trials published in recent years and applied subgroup analysis according to the characteristics of different studies. In addition, this review determined the effect of CS on health-related quality of life using the SGRQ, an important patient-reported outcome. This review used rigorous scientific method to assess the effect of CS on stable stage of COPD. Moreover, one point of difference between this and previous reviews is the analysis of different preparation types. We evaluated the effects of CS preparations and formulae separately. This allows greater translation to practice.

### 4.3. Pharmacological Activities and Clinical Research

*Cordyceps sinensis*, a rare crude herb, grows in high latitude areas in China. The fungus is parasitic on the insect [40]. The entomopathogenic fungus* Cordyceps s.l. (sensu lato)* species mainly includes* Ophiocordyceps sinensis* and* Cordyceps military* [41, 42]. In recent years, many studies have examined the pharmacological bioactivities of the herb and extracts of their active components. To date, CS has been well characterized as possessing abundant bioactive compounds, such as D-mannitol (cordycepic acid), cordycepin, adenosine, vitamins, polysaccharides, and enzymes [42]. Many studies demonstrate that the compounds in* Cordyceps military* have anti-inflammation activity [43], antioxidant activity [42, 44], antitumor activity [45, 46], and immune-regulatory function. CS preparations may offer potential advantages in the treatment of pulmonary disease.

In China, CS extract has been manufactured into preparations for easy administration. These include Jin Shui Bao capsule and Bai Ling capsule, whose indications for use are stable COPD, chronic kidney disease [47], hyperlipemia [48], and liver cirrhosis [49]. For stable COPD, CS preparations have been broadly used in clinical practice. The results from included studies of CS preparations show some potential benefits. However, methodology shortcomings in trial design and implementation have been identified, such as inappropriate randomization, no allocation concealment, and drug-misusing control. In recent years, the quality of clinical trial on CS has been improved to some extent. In this review, we search all the randomized controlled trials on CS up to December 2016 and include the patients of COPD of stages 2 to 3, who are the main applicable populations for CS treatment in China.

Among included studies, adverse events in people who received CS included dry mouth, abdominal distension, throat discomfort, and headache. Throat discomfort is a known adverse event of CS preparations and is listed on the product information sheet. Abdominal distension might be related to CS because it belongs to the category of TCM nourishment, patients with spleen deficiency may lead to indigestion. The rates of dry mouth and headache were very low, and they were probably not caused by CS. Based on the few adverse events reported in included studies, CS appear to be well tolerated.

### 4.4. Limitations and Advice on Future Research

In this review, none of the included studies provided calculation method of sample size nor discussed potential bias. Sample size was not more than 90 in any trial, and sample size in subgroups were often less than 100, so the reliability of the combined result is limited. In addition, seven of 14 studies, described the method of randomization, either by software or by random number table, two studies described allocation concealment, and bias of blinding participants was judged to be high in all the studies. Many studies reported no dropouts, which is surprising when the duration of the observation was up to 12 months. These methodological shortcomings above limit the conclusions of this review and highlight that further clinical studies on CS need methodology improvement. Besides the methodological shortcomings, the number of included studies in this review was small. This limited subgroup analysis and resulted in less certainty in the findings. The third limitation is that data were presented in aggregate for stage 2 and stage 3. As such, we were unable to compare the different effects when CS was used in different stages of COPD. Meanwhile, as CS is a Chinese herbal medicine, much of the evidence comes from the Chinese journals. This was expected. We intended to conduct analysis to assess publications bias; however, as there were fewer than 10 studies in comparisons for each outcome we were unable to do so. Publication bias is possible given that all included studies were conducted in China and published in Chinese language journals. Such studies may be more favourable toward CS. This may be another limitation of this manuscript. This review indicated that we need more rigorously designed clinical trials on CS. Besides, future trials of Chinese herbal medicines need to be properly registered prospectively in internationally recognized registries, to improve transparency in reporting.

## 5. Conclusion

This review found that* Cordyceps sinensis* could benefit patients with GOLD stage 2 to 3 COPD in terms of exercise tolerance and life quality. It may have a potential effect on improving lung function.* Cordyceps sinensis* seemed to be beneficial in reducing exacerbation and improving symptoms in stable COPD of GOLD stages 2 to 3 with few adverse events. But we could not draw strong conclusions on effectiveness or safety due to methodological weaknesses and risk of bias of the included studies.

## Figures and Tables

**Table 1:** Characteristics of included studies.

First author, year	No. of participants randomized/assessed; dropouts	Age; Gender (M/F)	COPD stage	Duration of condition (years)	Treatment duration; follow-up duration (months)
Bai SR, 2016	T:90/85;5	T:58.7(12.5);49/41	II,III	T:6.5(3.3)	T:6;0
	C:90/83;7	C:59.3(13.2):51/39		C:6.3(3.2)	C:6;0

Cao DC, 2016	T:50/50;0	T:66.33(4.21);30/20	II	NS	T:3;0
	C:50/50;0	C:64.45(5.11);28/22			C:3:0

Dong KZ, 2016	T:25/25;0	T:65.2(3.2);18/7	II,III	NS	T:3;0
	C:25/25;0	C:66.8(2.7);17/8			C:3:0

Fei DL, 2010	T: 45/45;0	T:59.7(4.5);28/17	II,III	T:10.5(4.9)	T: 3;0
	C: 45/45;0	C:57.9(4.1);26/19		C:11.5(5.2)	C: 3;0

He WJ, 2010	T: 24/22;2	T:55.36(9.03);15/7	II,III	T:12.22(2.75)	T: 3;0
	C: 24/23;1	C:57.13(8.77);17/6		C:11.56(3.08)	C: 3;0

Hu QG, 2012	T: 51/51;0	T:65.41(12.23);34/17	II,III	NS	T: 6;0
	C: 50/50;0	C:64.86(11.84);34/16			C: 6;0

Huang DH, 2005	T: 32/32;0	T:69.5(11.8);21/11	II,III	T:8.5(3.2)	T: 3;0
	C: 31/31;0	C:68.8(10.6);23/8		C:8.3(3.5)	C: 3;0

Liu JS, 2014	T: 40/40;0	Total:64.2(2.4);82/38	II,III	Total:15.2(3.9)	T: 12;0
	C: 40/40;0				C: 12;0

Luo SW,2015	T: 51/51;0	T:72.3(8.5);33/18	II,III	T:14.7(5.3)	T: 2;0
	C: 51/51;0	C:73.5(8.9);34/17		C:15.1(5.7)	C: 2;0

Tang CY, 2009	T: 30/30;0	T:63.2*∗*;27/3	II,III	T:7.2*∗*	T: 6;6
	C: 30/30;0	C:65.5*∗*;26/4		C:7.5*∗*	C: 6;6

Wu HL, 2016	T:46/46;0	T:66.58(7.12);31/15	II,III	T:5.82(1.64)	T:2;0
	C:32/32;0	C:66.34(7.09);21/11		C:6.03(1.71)	C:2;0

Xin DY, 2015	T: 41/41;0	T:58.6;21/20	II,III	T:10.2*∗*	T: 1;0
	C: 39/39;0	C:57.9;19/20		C:10.8*∗*	C: 1;0

Yao JF, 2013	T:30/27;3	T:67.48(6.99);18/9	III	T:6.33(4.01)	T: 3;0
	C:30/25:5	C:67.68(7.76);18/7		C:4.68(3.16)	C: 3;0

Zhou Y, 2014	T:50/50;0	T:62.8(6.2);29/21	II	T:14.5(8.5)	T: 6;6
	C:50/50;0	C:61.6(7.4);30/20		C:13.5(9.5)	C: 6;6

*∗* median.

**Table 2:** Intervention in included studies.

First author, year	Intervention	Control
Bai SR, 2016	Bai Ling Capsule: Dong Chong Xia Cao, 3 capsules, tid; Bu Fei Huo Xue Capsule, 4 Capsules, tid; Salmeterol/fluticasone, 50/250 ug, bid; or add aminophylline, 0.1 g, bid; Oxygen therapy	Salmeterol/fluticasone, 50/250 ug, bid; or add aminophylline, 0.1 g, bid; Oxygen therapy

Cao DC, 2016	Bai Ling Capsule: Dong Chong Xia Cao, 5 capsules, tid; routine care	Routine care*∗*

Dong KZ, 2016	He Che Chong Cao Capsule: Dong Chong Xia Cao, Zi He Che, Hong Shen, San Qi, Ge Jie, Lu Jiao Shuang, Bei Mu, 4 Capsules, tid; budesonide/formoterol, 60 ug/4.5 ug, bid	Budesonide/formoterol,60 ug/4.5 ug, Bid

Fei DL, 2010	Jin Shui Bao Capsule: Dong Chong Xia Cao; routine care	Routine care

He WJ, 2010	Bai Ling Capsule: Dong Chong Xia Cao, 4 capsules, tid; Shan Yu Rou, Yin Yang Huo, Wu Wei Zi, Tai Zi Shen, Bai Guo, Fu Ling, Dan Shen; routine care	Routine care

Hu QG, 2012	Jin Shui Bao Capsule:Dong Chong Xia Cao, 3 capsules, tid; Bu Fei Huo Xue Capsule, 4 Capsules, tid; Oxygen therapy, if AECOPD: antibiotics, aminophylline, anticholinergic, ICS	Oxygen therapy, if AECOPD: antibiotics, aminophylline, anticholinergic, ICS

Huang DH, 2005	Bai Ling Capsule: Dong Chong Xia Cao, 5 capsules, tid; Yu Ping Feng Powder: Huang Qi, Bai Zhu, Fang Feng, 5 g, tid; Jian Pi Yi Fei Powder: Ren Shen, Bai Zhu, Fu Ling, Mai Dong, Sang Bai Pi, Huang Qi, 10g, tid; routine care	Routine care

Liu JS, 2014	Jin Shui Bao Capsule:Dong Chong Xia Cao, 3 capsules, tid; Yu Ping Feng Powder: Huang Qi, Bai Zhu, Fang Feng, 6 g, tid; routine care	Routine care

Luo SW, 2015	Bai Ling Capsule: Dong Chong Xia Cao, 5 capsules, tid; routine care	Routine care

Tang CY, 2009	Bai Ling Capsule: Dong Chong Xia Cao, 3 capsules, tid; routine care	Routine care

Wu HL, 2016	Bai Ling Capsule: Dong Chong Xia Cao, 5 capsules, tid; routine care	Routine care

Xin DY, 2015	Li Fei tablet: Dong Chong Xia Cao, Ge Jie, Wu Wei Zi, Bai He, Bai Bu, Gan Cao, Pi Pa Ye, Bai Ji, etc., 2 tablets, tid; aminophylline, 0.2 g, bid; Ambroxol hydrochloride, 30 mg, tid.	Aminophylline, 0.2 g, bid; Ambroxol hydrochloride, 30 mg, tid.

Yao JF, 2013	Dong Chong Xia Cao, Huang Qi, Hong Jing Tian, Yin Xing, Gua Lou, Hai Ge Qiao, Shui Zhi, Ma Huang; Salmeterol/fluticasone, 50/250 ug, bid; aminophylline, 0.1 g, bid	Salmeterol/fluticasone, 50/250 ug, bid; aminophylline, 0.1 g, bid

Zhou Y, 2014	Bai Ling Capsule: Dong Chong Xia Cao, 5 capsules, tid; routine care; salmeterol/fluticasone, 50/250 ug, bid	Routine care; salmeterol/fluticasone, 50/250ug, bid

^*∗*^The treatments follow therapeutic principle in gidelines, but the interventions may be different base on patients' conditions.
